# Detection by Direct Next Generation Sequencing Analysis of Emerging Enterovirus D68 and C109 Strains in an Environmental Sample From Scotland

**DOI:** 10.3389/fmicb.2018.01956

**Published:** 2018-08-21

**Authors:** Manasi Majumdar, Javier Martin

**Affiliations:** Division of Virology, National Institute for Biological Standards and Control, Hertfordshire, United Kingdom

**Keywords:** human enterovirus, enterovirus pathogenesis, environmental surveillance, next generation sequencing, EV-D68, EV-C109, direct detection

## Abstract

**Background:** Human enteroviruses (EVs) have been linked with severe disease and syndromes as varied as acute respiratory illness, myocarditis, and flaccid paralysis. With global polio eradication on sight the focus of clinical investigations has expanded to the identification of other EV serotypes associated with severe neurological conditions such as EV-D68, responsible for large outbreaks in 2014 and 2016 that spread worldwide and were related with severe respiratory disease leading to acute myelitis in some cases. New EV serotypes with epidemic potential continue to emerge such as EV-C104, EV-C105, EV-C109, and EV-C117 identified in respiratory samples in recent years.

**Methods:** We used a next generation sequencing (NGS) approach to detect multiple EV serotypes directly in a sewage concentrate from Glasgow (Scotland, United Kingdom) generating whole-capsid nucleotide sequences that were compared to sequences of cell culture isolates from this sewage sample and clinical EV isolates from GenBank.

**Results:** Thirteen different serotypes belonging to all four A, B, C, and D EV species were identified in the sewage concentrate. EV strains closely related to EV-D68 epidemic isolates of B3 lineage reported in the United States and Europe in 2016 and to EV-C109 respiratory isolates found in Denmark and Netherlands in 2015 were identified.

**Conclusion:** Environmental surveillance (ES) can effectively detect EV circulation in human populations. The use of NGS for ES can help overcoming the limitations of traditional cell culture and sequencing methods, which are selective and biased, and can contribute to the early detection and assessment of spread of emerging EV pathogens.

## Introduction

Human enteroviruses (EVs) are common human pathogens that have been associated with severe disease including syndromes such as acute respiratory disease, meningitis, myocarditis, hepatitis, and flaccid paralysis, the most notorious being paralytic poliomyelitis caused by poliovirus (PV) (Tapparel et al., 2013). EVs belong to the family Picornaviridae and are classified into four species (EV-A to EV-D) based on their genetic identity with 110 different EV serotypes known at present. The non-enveloped virus particles contain a single-stranded positive-sense RNA genome about 7,500 nucleotides in length that contains a single open reading frame coding for structural (VP1 to VP4 capsid proteins) and non-structural proteins (2A to 2C and 3A to 3D), flanked by 5′ and 3′ non-coding regions. The focus of clinical investigations in recent years has expanded to the identification of EV serotypes other than PV associated with severe neurological syndromes. EV-D68 has been linked with severe respiratory disease, occasionally associated with acute flaccid myelitis (AFM). A large outbreak of severe respiratory illness and few AFM cases associated with EV-D68 infection occurred in the United States starting in August 2014. Genetically related EV-D68 strains were also found during the same period in Canada, Europe, and Asia with more than 2,000 cases reported in 20 countries (Holm-Hansen et al., 2016). These outbreaks were temporally and geographically associated with clusters of increased AFM, particularly in the United States. Among the people confirmed with AFM, EV-D68 was not consistently detected in every patient (Holm-Hansen et al., 2016). However, there is growing evidence of the association of AFM with severe respiratory disease and EV-D68 infection in a number of countries. An outbreak of EV-D68, caused by new lineage B3 strains, was reported in Sweden in August–September 2016 with 74 cases identified (Dyrdak et al., 2016). Ten patients had severe respiratory or neurological symptoms and one died. This outbreak coincided in time with an upsurge of isolations of related strains between June and September 2016 reported in other European countries and the United States, which correlated with an increase in EV-related severe infections (Pariani et al., 2017; Wang et al., 2017). Two recent meta-analysis studies published independently concluded that, while more research is needed on some aspects such as a dose–response relationship, application of the Bradford Hill criteria, a set of nine principles applied to examine causality, supported a causal relationship between EV-D68 and AFM (Dyda et al., 2018; Messacar et al., 2018).

New EV serotypes have continued to emerge in recent years notably EV-C respiratory isolates associated with mild to moderately severe respiratory disease and occasional neurological complications. In particular, EV-C109 was first identified in respiratory samples from Nicaragua in 2008 (Yozwiak et al., 2010) and was since reported in earlier respiratory samples from Italy in 2005, Hungary in 2007, and Denmark in 2007 (Debiaggi et al., 2012; Pankovics et al., 2012) and, more recently, in respiratory samples from Netherlands (Van Leer-Buter et al., 2016) and Denmark (Barnadas et al., 2017) following enhanced surveillance. Other members of the EV-C species were first found in respiratory samples such as C-104 found in Switzerland in 2005 in samples from patients with respiratory illnesses (Tapparel et al., 2009) and C117 found in a Lithuanian child suffering from pneumonia (Daleno et al., 2012). EV-C105 was first identified in the Democratic Republic of Congo in late 2010 in a fecal sample collected from a fatal case during a poliomyelitis outbreak (Lukashev et al., 2012). Since then, EV-C105 was also identified in a patient in Peru (Tokarz et al., 2013) and in another patient in Cyprus (Richter et al., 2013), both with respiratory disease. Although only few isolates of these serotypes exist and their incidence appear to be low, the reported isolations suggest a global distribution. Therefore the potential for outbreaks due to respiratory EVs is a concern. However, EV infections are usually not tested or treated so the number of EV detections is likely a substantial underestimate of the true burden of disease. Furthermore, most of these respiratory EV strains appear not to be present in stools (Van Leer-Buter et al., 2016; Barnadas et al., 2017), which are the clinical samples most commonly tested in many countries, as they are the samples used for PV surveillance. Consequently, sequence information of these new EV strains is sporadic and biased probably not reflecting their true genetic diversity. A new European non-polio enterovirus network (ENPEN) has recently been established with an aim to improve diagnostics for EVs, collect data on severe EV infections and monitor the circulation of different EV serotypes (Harvala et al., 2018). Recommendations on the most adequate samples to be analyzed and methods for non-polio EV detection and typing were made. The final goal is to establish standardized surveillance systems that will be used to provide a better estimation of EV disease burden. In addition to clinical diagnosis, environmental surveillance (ES) for PV has proven to be a very sensitive method to detect PV circulation, even in the absence of paralytic disease, and has greatly helped tracing the elimination of wild and vaccine PV in different regions (Asghar et al., 2014). There is also evidence that EV serotypes from all four A, B, C, and D species are commonly present in sewage samples from different countries (Ndiaye et al., 2014) and therefore ES could also be useful to estimate the prevalence and circulation patterns of non-polio EVs in human populations.

We have analyzed an environmental sample taken in Scotland for the presence of EV strains from different serotypes. We used a next generation sequencing (NGS) approach with whole-capsid RT-PCR products directly amplified from the sewage concentrate and compared the results with sequences of EV isolates from cells infected with the sewage concentrate and other EV sequences from GenBank. There are generally no marked seasonal trends in EV infections in the United Kingdom although characteristic peaks in the summer and troughs in the winter have been observed during periods of high incidence (Kadambari et al., 2014). We show the value of using direct NGS analysis for the detection of multiple EV strains from all four A to D species in sewage, including EV serotypes typically found in respiratory samples.

## Materials and Methods

### Sewage Sample Collection and Processing

A 1 L grab sewage sample was collected at random from the inlet of Shieldhall sewage plant in Glasgow (Scotland), with a catchment area covering 585,000 people, on November 25, 2015. The sample was frozen prior to being processed and concentrated by filtration and centrifugation using a Centriprep YM-50 centrifugal concentration device (Merck) as described before (Harvala et al., 2014). Briefly, the crude sewage sample was clarified by centrifugation at 3,000 *g* and 21°C for 10 min. The supernatant was then passed through a 0.45 μm filter (Merck). The filtrate was loaded on to a YM-50 Centriprep^TM^ Centrifugal Filter (Merck) and centrifuged at 1,500 *g* and 21°C for 20 min followed by 15 min at 1,500 *g* and 21°C to collect the retained material. A volume of around 4 ml was obtained from the 120 ml of crude starting sewage material, thus attaining a concentration factor of 30×.

### Virus Isolation in Cell Culture

Virus isolation in cell culture was performed according to WHO recommendations (World Health Organization, 2004). Human rhabdomyosarcoma (RD) cells were used for this purpose.

### Pan-EV Entire Capsid-Coding Region RT-PCR Amplification

We followed a method designed to amplify entire-capsid sequences of EV strains from all four A, B, C, and D species modified from a method described before, which was designed to primarily amplify PV sequences (Arita et al., 2015). Following analysis of enterovirus nucleotide sequences from GenBank, two independent PCR aimed at amplifying all known enterovirus strains were used. The products from the two PCR were mixed and analyzed in a single NGS reaction. Viral RNA was extracted from the sewage concentrate using Roche High Pure viral RNA kit. RT-PCR fragments covering the entire capsid-coding region and part of the region coding for non-structural proteins (2A–2C) were synthesized from purified viral RNAs by one-step RT-PCR using a SuperScript III One-Step RT-PCR System with Platinum Taq High Fidelity DNA Polymerase (Invitrogen). Two different reactions were performed using two primer sets:

a. Primers 5′NCR (5′-TGGCGGAACCGACTACTTTGGGTG-3′) and CRE-R (5′-TCAATACGGTGTTTGCTCTTGAACTG-3′)
b. Primers MM_EV_F2 (5′-CAGCGGAACCGACTACTTT-3′) and MM_EV_R1 (5′-AATACGGCATTTGGACTTGAACTGT-3′)

Reaction conditions were: 50°C for 30 min followed by 94°C for 2 min plus 42 cycles of 94°C for 15 s, 55°C for 30 s and 68°C for 8 min with a final extension step of 68°C for 5 min. Amplified products from both reactions were purified using AMPure XP magnetic beads (Beckman Coulter) and pooled (1:1) before being analyzed by NGS. The expected amplicon size for both RT-PCR is approximately 4,000 nucleotides (nucleotides 553–4,459, numbering as in PV1 Sabin AY184219 reference strain).

### Generation of Sequencing Libraries and Quality Trimming of NGS Reads

**FIGURE 1:**
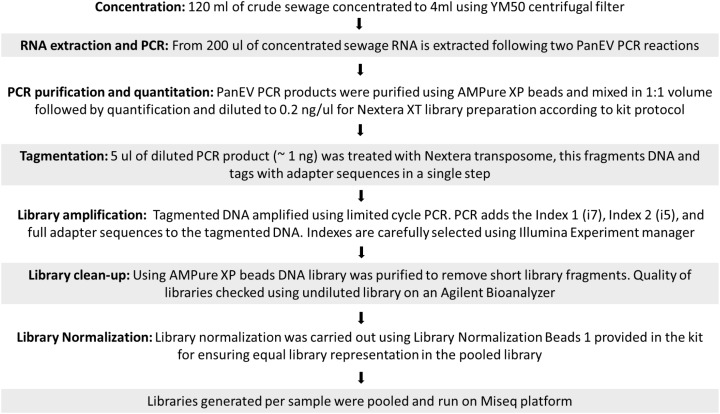
Flowchart of sequence library preparation.

Sequencing libraries were prepared using Nextera XT reagents and sequenced on a MiSeq using a 2 × 301-mer paired-end v3 Flow Cell and manufacturer’s protocols (Illumina). NGS sequencing data were analyzed using the Geneious R10 software package (Biomatters) as described before (Majumdar et al., 2017). A flowchart describing the different steps used from raw sewage concentration to sequence library preparation is shown in **Figure 1**. Raw sequence data were then imported into Geneious and paired end reads combined. Data were filtered using a custom workflow with the following parameters: PCR primers and Nextera adaptor/index sequences were trimmed from 5′ and 3′ ends with a minimum 5 bp overlap; reads were trimmed to have no bases with a quality <Q30 and no ambiguities. Following this, reads <50 nt in length were discarded and duplicate reads were removed using the program Dedup (within Geneious).

### Generation of EV Sequence Contigs by *de novo* Assembly of Filtered NGS Reads

The filtered NGS reads were assembled *de novo* using stringent assembly conditions as described before (Majumdar et al., 2017): minimum 50 base overlap, minimum overlap identity of 98%, maximum 2% mismatches per read and only using paired hits during assembly. In addition, the options to produce scaffolds and ignore words repeated more than 100–1,000 times, available in the Geneious assembler, were selected to improve the quality of assembly. Following BLAST analysis, only contigs with mean coverage >30 nt per site and covering the capsid coding region were selected for further analysis. Filtered reads were finally mapped to these selected contigs to obtain final consensus sequences by assigning the most common nucleotide sequence to each nucleotide position. Manual analyses for visualizing and quantifying assembly results were performed throughout the process. As a result, we determined nucleotide sequences for a number of EV strains in each sample. The filtered NGS reads were also analyzed using VirusTAP: Viral Genome-Targeted Assembly Pipeline, available online (Yamashita et al., 2016). Raw fastq files were submitted online and three different *de novo* assembly algorithms were used: SPAdes, IDBA-PriceTI, and A5-miseq using VirusTAP default settings. The results were compared to those obtained with our Geneious pipeline.

### Identification of EV Serotypes in the Sewage Concentrate and Infected Cell Extract

The closest virus relatives to each of the sewage EV final consensus sequences were identified using the RIVM and BLAST online sequence analysis tools and EV serotypes were assigned based on their VP1 sequence.

### Nucleotide Sequence Analysis of the VP1 Coding Region of EV Strains by the Sanger Method

VP1 nucleotide sequences of the D68 and C109 strains identified in the sewage sample from Scotland were also analyzed by Sanger sequencing. RT-PCR fragments containing VP1 sequences were generated from purified Pan-EV RT-PCR products by PCR using a Platinum Taq High Fidelity Enzyme (Invitrogen) system and specific primers for each serotype. Amplified products were purified using QIAquick PCR purification kit (Qiagen,) and sequenced using an ABI Prism 3130 genetic analyzer (Applied Biosystems).

### Phylogenetic Analysis of EV Strains

Enterovirus sequences obtained in this study were compared to those of other EVs available in the GenBank database. EV genome sequences were aligned using the program ClustalW (within Geneious). Molecular Evolutionary Genetics Analysis (MEGA) software package version 7.0 (Kumar et al., 2016) was used for phylogenetic analyses. The evolutionary history of aligned sequences was inferred using the neighbor-joining method with evolutionary distances computed using the maximum composite likelihood substitution method. Sequence divergence was determined by calculating mean pairwise distances.

### Bayesian Phylogenetic History Analysis of EV-D68 Strains

A Bayesian Markov chain Monte Carlo (MCMC) analysis using BEAST version 1.8.2 software (Drummond et al., 2012) was also used to assess the evolutionary history of EV-D68 VP1 sequences. The general time reversible (GTR) model of nucleotide substitution with invariant sites was used. Two independent chains of 50 million steps each were run under the uncorrelated lognormal distribution clock model. The collection dates were included as temporal data. Effective sample size values were monitored for consistency using Tracer v1.6^1^ and convergence was easily obtained. Maximum clade credibility (MCC) trees were calculated with the TreeAnnotator version 1.8.2 program^2^, and estimates of posterior probability density were obtained for each node. Phylogenetic trees were drawn using FigTree version 1.4.2 program^3^.

## Results

### Sample Processing, Virus Isolation, and Preparation of Pan-EV Entire Capsid-Coding Region RT-PCR Products for NGS Analysis

A total of 120 ml of raw sewage were processed as described in Section “Materials and Methods.” A final volume of approximately 4 ml of sewage concentrate was recovered and a 0.5 ml sample of this concentrate was used to infect RD cells on a 25 cm^2^ flask. Cells showed full CPE after 4 days incubation at 35°C and cell debris and supernatant were collected and stored -20°C for further processing. RT-PCR products covering the entire EV capsid-coding region were generated with primers designed to amplify all known EV strains from all four A, B, C, and D EV species.

### Identification by NGS of EV Strains Present in the Sewage Concentrate and the Infected Cell Extract

**FIGURE 2:**
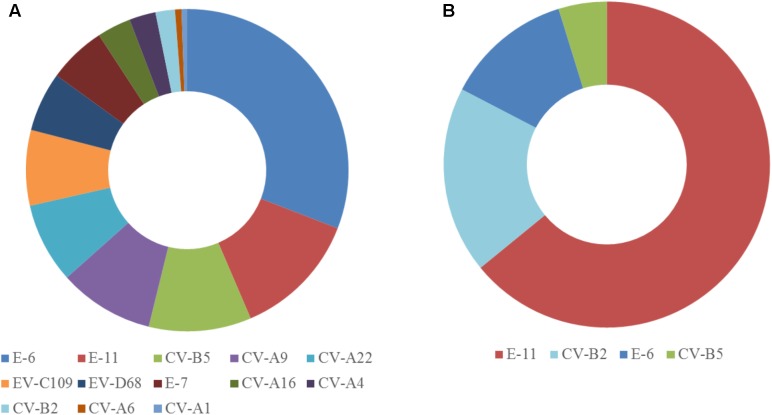
Enterovirus serotypes identified in the sewage concentrate and infected cell extract. Consensus sequences were obtained as described in Section “Materials and Methods.” Filtered NGS reads from RT-PCR products obtained from RNA purified from the sewage concentrate **(A)** or the infected cell extract **(B)** were mapped against consensus sequences obtained for each sample. The proportion of reads mapping each EV serotype are shown.

The two samples were analyzed in a MiSeq run containing a total of 90 samples, including negative controls and unrelated samples. NGS reads from Pan-EV RT-PCR products amplified from viral RNAs extracted from the sewage concentrate and the infected cell extract were filtered and analyzed as described in Section “Materials and Methods.” A total of 360,388 and 437,314 filtered reads for the sewage concentrate and the infected cell extract were sequenced in our study, respectively. A high proportion of the total filtered NGS reads from both samples mapped to final EV contig consensus sequences (93.7 and 95.3%, respectively). A total of 20 contig sequences belonging to 13 different EV serotypes including strains from all four A, B, C, and D species were identified in the sewage concentrate whereas strains very closely related to four of these EV concentrate strains (from serotypes E-11, CV-B2, E-6, and CV-B5) were identified in the infected cell extract (**Figure 2**). E-11, E-6, and CV-B5 were the most abundant strains in the sewage concentrate but CV-B2 viruses were present in lower proportion. No EV-A, EV-C, or EV-D strains were found in the infected cell extract. **Table 1** shows the genetic properties of the EV sewage strains in comparison to their closest relatives from the GenBank database. EV strains genetically close to most EV sewage strains found here have been reported from across the world.

**Table 1:** Genetic properties of EV strains identified in the sewage concentrate by NGS.

Contig No.	No. of reads (mean coverage)^1^	Genome coverage^2^	Serotype^3^ (cell isolate)	Closest relative from GenBank database		
				Accession No.	%Identity^4^	Location, year
1	1,902 (105×)	584–4,400	CV-A1	KF412999	96.9	India, 2009
2	5,227 (273×)	562–4,441	CV-A4	KR185978	91.6	Russia, 2013
3	3,717 (194×)	590–4,440	CV-A4	KR185978	91.3	Russia, 2013
4	2,193 (115×)	570–4,438	CV-A6	KX212501	98.3	Thailand, 2014
5	32,265 (4,274×)	555–4,431	CV-A9	KY674976	98.9	United States, 2016
6	8,342 (345×)	562–4,420	CV-A16	LT577809	99.2	France, 2014
7	3,101 (161×)	582–4,419	CV-A16	LT617113	99.2	France, 2014
8	12,481 (887×)	550–4,421	CV-A22	KP747504	99.3^5^	Russia, 2012
9	10,595 (449×)	641–4,420	CV-A22	KF984203	98.8	Italy, 2013
10	4,087 (238×)	573–4,005	CV-A22	KY909307	98.2	Thailand, 2011
11	6,583 (334×)	557–4,385	CV-B2 (99.9)	KU557321	95.6	China, 2011
12	34,685 (1,548×)	553–4,382	CV-B5 (99.9)	MG451803	99.4	United Kingdom, 2016
13	20,117 (1,026×)	725–4,420	E-11	KP090690	96.7	Russia, 2012
14	3,699 (185×)	557–4,431	E-11	LC361292	96.5	China, 2016
15	19,001 (971×)	560–4,418	E-11 (100)	KU133585	97.4	Russia, 2012
16	83,659 (1,844×)	547–4,407	E-6 (99.3)	HG793688	97.7	France, 2011
17	20,563 (1,325×)	1,337–4,397	E-6	HG793688	97.7	France, 2011
18	19,571 (1,028×)	557–4,406	E-7	LC062742	97.9	Japan, 2014
19	25,567 (1,351×)	553–4,380	EV-C109	KT735360	99.7	Netherlands, 2015
20	20,155 (1,104×)	554–4,390	EV-D68	KX675262	99.0	United States, 2016

*^1^Mean sequence coverage per nucleotide position; ^*2*^nucleotide genomic position; ^*3*^GenBank accession numbers: MH361013–MH361032. In brackets, % of nucleotide sequence identity between EV strain from concentrate and EV cell isolate present in infected cell extract; ^*4*^VP1 nucleotide sequence identity except ^*5*^VP4–VP2 sequences compared*.

Enterovirus contig sequences were also constructed using the VirusTAP online tool. The results using three different *de novo* assembly algorithms are shown in **Table 2**. Minor differences were observed between assembly methods, particularly for contigs with small number of reads as some methods are less sensitive. There are also slight differences between methods in how contigs for related sequences (genetically related variants from the same serotype in this case) are built up. However, results overall were very similar to those obtained using our Geneious pipeline producing nearly identical consensus sequences. Negative control samples including RNA extraction, RT-PCR and water controls were also sequenced and did not contain relevant EV sequences above background levels. Complete capsid sequences of all EV strains identified in the sewage concentrate have been deposited at GenBank (NCBI accession numbers MH361013–MH361032).

**Table 2:** Comparison of *de novo* assembly results described in this study to those obtained using the VirusTAP online application (Yamashita et al., 2016).

Contig No.^1^	IDBA-PriceTI		A5-miseq		SPAdes	
	
	GC (%)^2^	SI (%)^3^	GC (%)	SI (%)	GC (%)	SI (%)
1					85.1	100
2	92.6	100	53.7	100		
3	52.1	100	4.2	100		
4	59.4	99.9			63.3	100
5	93.2	99.5	94.7	99.8	95.7	100
6	97.6	99.8	84	100	98.4	100
7	28.7	100			69.5	100
8	40.4	99.5	73.4	99.9	95.3	100
9	46.6	100	27.4	100	26.3	100
10	91.1	99.5	49	100	81.1	100
11	94.3	100	82.6	100	25.5	100
12	80.1	99.8	91	100	96.2	100
13	99.5	100	94.6	100	96.3	100
14	10.3	100	99	99.7		
15	98.1	100	93.7	100	90.9	100
16	86.3	99.8	89.6	99.9	87.3	100
17	21	99.5	68.8	100	99.2	100
18	96.2	100	94.1	100	82.2	100
19	67.8	99.6	84.9	99.1	91.8	100
20	96.7	100	96.2	100	92.1	100

*^1^Contig number as in **Table 1**. Empty cells indicate no similar contig was found using that particular method. ^*2*^GC: Genome coverage of consensus sequences generated by VirusTAP relative to results shown in **Table 1**. ^*3*^SI: Sequence identity of consensus sequences generated by VirusTAP relative to results shown in **Table 1***.

### Genetic Characterization of EV-C109 and EV-D68 Sewage Strains From Scotland

EV-C109 and EV-D68 strains identified in sewage concentrates were further characterized. A total of 20,204 and 25,622 NGS reads mapped to EV-C109 and EV-D68 sequences, respectively, showing very even coverage across the sequenced region (data not shown). VP1 sequences were also determined by the Sanger method showing identical results to those found by NGS. We observed mixed peaks at some nucleotide positions in the Sanger sequence electropherograms, mostly at third-base synonymous sites. These mixed bases were also detected as single nucleotide polymorphisms (SNPs) in the NGS contig assemblies and most likely reflect the presence of virus variants (data not shown).

**FIGURE 3:**
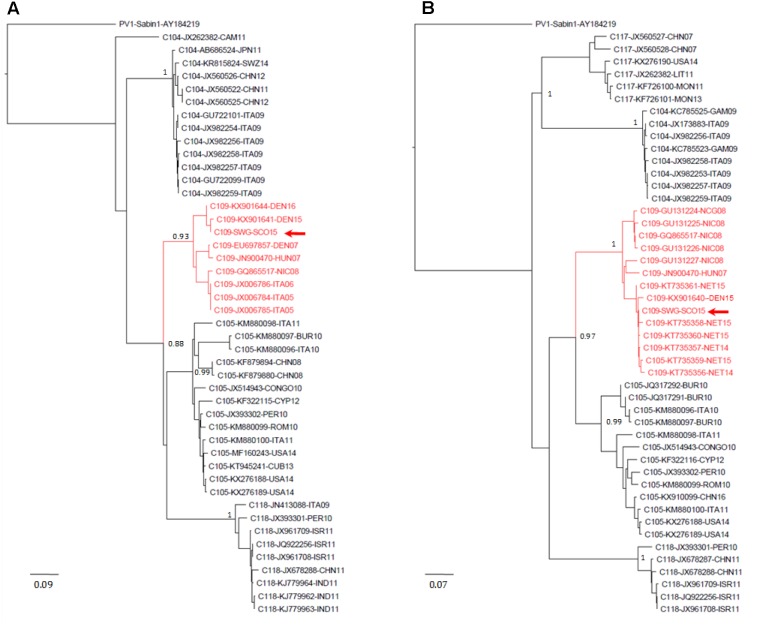
Evolutionary relationships of Enterovirus C109 strains. The evolutionary histories were inferred using the neighbor-joining method. Results for VP4–VP2 **(A)** and VP1 **(B)** sequences are shown. The optimal trees with the sum of branch length = 2.54566021 **(A)** or = 3.00774278 **(B)** are shown. The percentage of replicate trees in which the associated taxa clustered together in the bootstrap test (1,000 replicates) are shown next to the branches. The trees are drawn to scale, with branch lengths in the same units as those of the evolutionary distances used to infer the phylogenetic trees. The evolutionary distances were computed using the maximum composite likelihood method and are in the units of the number of base substitutions per site. The rate variation among sites was modeled with a gamma distribution (shape parameter = 4). The analysis involved 46 **(A)** and 54 **(B)** nucleotide sequences. All ambiguous positions were removed for each sequence pair. There were a total of 423 **(A)** and 899 **(B)** nucleotide positions in the final datasets. Accession number, location, and year of isolation are indicated in the sequence names. EV-C109 strain from the Scottish sewage sample is indicated with a red arrow. Evolutionary analyses were conducted in MEGA7 (Kumar et al., 2016). Abbreviations for country names are: ITA, Italy; USA, United States; CHN, China; THA, Thailand; SWZ, Switzerland; DEN, Denmark; CYP, Cyprus; CUB, Cuba; PER, Peru; ROM, Romania; ISR, Israel; IND, India; NIC, Nicaragua; CAM, Cameroon; BUR, Burundi; NET, Netherlands; HUN, Hungary; LIT, Lithuania; GAM, Gambia; MON, Mongolia.

**FIGURE 4:**
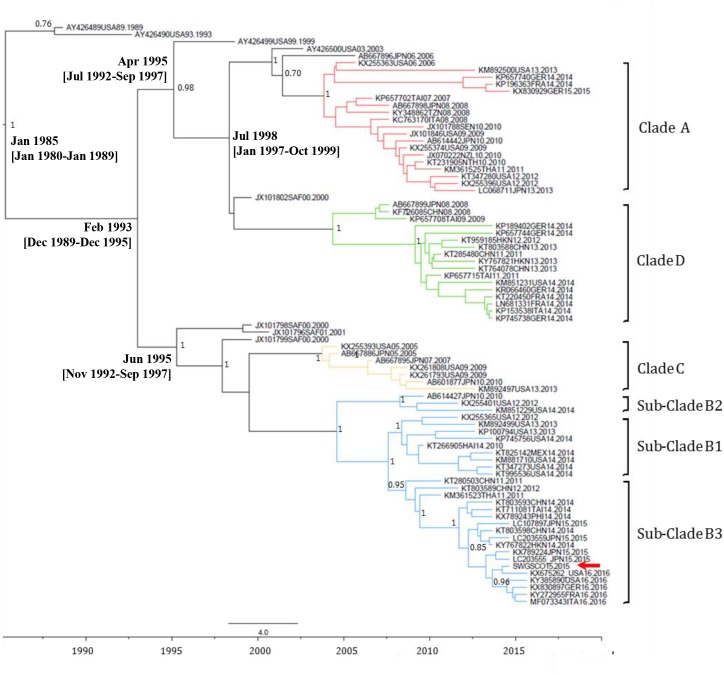
Maximum clade credibility (MCC) phylogenetic tree of EV-D68 VP1 sequences. The analysis involved 48 nucleotide sequences. There were a total of 892 nucleotide positions in the final dataset. The scale indicates calendar years. The posterior probability is indicated for relevant nodes. Branches are colored according to genetic clade. Names for phylogenetic groups (clades and sub-clades) were assigned as in Gong et al. (2016). Accession number, location, and year of isolation are indicated in the sequence names. The position of the EV-D68 strain from the Scottish sewage sample is indicated with a red arrow. Estimated branch ages with 95% highest probability density (HPD) values are indicated for relevant nodes. Analysis was performed using BEAST version 1.8.2 software (Drummond et al., 2012). Abbreviations for country names are: ITA, Italy; USA, United States; MEX, Mexico; JPN, Japan; CHN, China; THA, Thailand; GER, Germany; SAF, South Africa; NZL, New Zealand; TAI, Taiwan; NTH, Netherlands; SEN, Senegal; TZN, Tanzania; FRA, France.

EV-C109 sewage strain sequences were closely related (99.7% sequence identity) to VP1 sequences from 2015 EV-C109 isolates from Netherlands and VP4 sequences (98.8% sequence identity) from a 2015 EV-C109 isolate from Denmark (**Figure 3**). The EV-D68 sewage strain from Scotland clustered within genetic sub-clade B3 and was genetically linked (99.0% VP1 sequence identity) to 2016 EV-D68 isolates from the United States and also related to other EV-D68 strains isolated in Europe in 2016, preceding multiple related clinical virus isolations. This EV-D68 strain found in the Scottish sewage was also genetically linked to 2014-2015 EV-D68 isolates from Japan and China from where this sub-clade B3 might have been originated (**Figure 4**).

## Discussion

We report the direct detection by NGS of EV strains from 13 different serotypes in a sewage sample from Scotland retrieving entire capsid coding sequences. The most prevalent strains belonged to species B serotypes but EV strains from species A (CV-A4, CV-A6, and CV-A16), C (CV-A1, CV-A22, and EV-C109), and D (EV-D68) serotypes were also found. Previous studies have shown a high frequency of EV-B strains in Europe and the United States with the occasional expansion of EV-A and EV-D serotypes such as CV-A6, CV-A16, EV-A71, or EV-D68 (Benschop et al., 2017; Brinkman et al., 2017). However, a high number of EV-C strains in sewage samples have been reported before in Scotland using direct PCR with EV species-specific primers (Harvala et al., 2014). The lack of detection of EV-C strains in clinical samples has been previously noted but it is not completely understood. Although the presence of EV virus strains from human excretions in sewage reflect their presence and circulation in humans, this does not necessarily mean that sewage is a source of EV infections. The potential of sewage causing EV infections in humans is expected to vary significantly between different geographical regions depending on wastewater infrastructures, general hygiene and other factors like climate.

Our study highlights the value of using NGS for the characterization of environmental samples containing complex EV mixtures giving a level of detail not possible with traditional cell culture and Sanger sequencing methods. The use of NGS methodologies over the last several years has resulted in a massive increase in our ability to sequence viral genomes. These methodologies have been used for multiple virology applications such as virus discovery (Palacios et al., 2008), clinical diagnostics (Prachayangprecha et al., 2014), virus surveillance (Kapoor et al., 2009; Baillie et al., 2012; Fernandez-Cassi et al., 2018), detection of drug-resistance minority variants in HIV (Palmer et al., 2005) and HCV (Verbinnen et al., 2010), and detection of an adventitious virus in a live-attenuated viral vaccine (Victoria et al., 2010).

Next generation sequencing techniques for sequencing viral RNA molecules in biological samples can be generally divided in methods using randomly generated RT-PCR products, e.g., those obtained by sequence-independent single-primer amplification (SISPA) (Allander et al., 2001; Kapoor et al., 2008) and methods using RT-PCR products generated with specific primers. The former type of methods have the advantage of having no strong bias for any particular sequence giving an accurate representation of all the RNA sequences present in a particular sample. These methods are useful when sequencing viral genomes present in high abundance in the sample such as those in infected cell extracts or when a very large number of reads are sequenced in a single sample. The later methods, as the one used in our present study, ensure that a high proportion of reads will correspond to sequence/s of interest and they are more suitable when sequencing viral genomes present in low proportion in the sample, such as EVs in sewage concentrates. NGS methods sequencing RNA molecules directly have similar characteristics to random RT-PCR methods but with the added benefit of not requiring RT-PCR amplification steps that might introduce errors in the sequence. NGS metagenomics and target-specific techniques have been described before to obtain nucleotide sequences of EV strains present in clinical samples, either directly from the sample or from infected cell cultures (Victoria et al., 2009; Yozwiak et al., 2010; Huang et al., 2015; Bessaud et al., 2016; Midgley et al., 2017; Montmayeur et al., 2017; Fernandez-Garcia et al., 2018).

Enteroviruses, together with multiple other known and new RNA and DNA viruses of diverse origins, have been identified by NGS in untreated sewage samples (Ng et al., 2012). In addition, two recent studies reported the whole-genome characterization of polio and non-polio EVs in mixtures present in cell cultures infected with sewage concentrates (Furtak et al., 2016; Majumdar et al., 2017). Earlier advances in the detailed characterization of the diversity of adenovirus, norovirus, and astrovirus by NGS analysis of short PCR amplicons obtained directly from sewage concentrates have been reported (Ogorzaly et al., 2015; Prevost et al., 2015; Kazama et al., 2016). The studies showed that these amplicon NGS assays can reveal more details of the distribution of viral genotypes in human populations than conventional approaches and support the idea of using sewage samples to analyze the global circulation of enteric viruses. However, EV sequence information from direct NGS analyses of sewage concentrates is still very limited. A recent study used NGS to determine the taxonomic distribution and seasonal dynamics of EVs in Ohio (United States) during a 1-year using two gene targets coding for capsid proteins VP1 and VP4, and found sequences matching as many as 85 different EV serotypes and showing differences in the seasonal distribution of EV serotypes from different species (Brinkman et al., 2017). Short VP1 amplicons (340 nt), typically used for typing enteroviruses in clinical samples, were used and found to be inefficient for targeting EVs in sewage. The authors argued that this was likely due to the high degeneracy of the VP1 primers used that resulted in cross-reactivity with unknown bacteriophages present in the complex background of sewage. The method used in our study produces much longer nucleotide sequences for each EV strain (approximately 4,000 nt) covering the entire capsid region and part of the region coding for non-structural proteins, hence allowing in-depth phylogenetic analyses to establish temporal and geographical links between sewage strains from different locations and between sewage strains and clinical isolates. The primers used in our study showed very high specificity for enterovirus sequences as 93.7 and 95.3% of the total filtered NGS reads for the sewage concentrate and the infected cell extract mapped to EV strains, respectively. In addition, a low number of filtered reads (between 1,077 and 17,516 per sample) were sequenced in the Ohio study versus the 360,388 and 437,314 filtered reads for the sewage concentrate and the infected cell extract sequenced in our study, again limiting the number of different amplicons that were sequenced and identified per sample.

This technique can also be used to type and sequence enterovirus strains present in clinical samples and/or cell culture isolates as current molecular methods are limited and only produce short nucleotide sequences of the most abundant strain/s present in samples. In addition, methods used to detect and type enterovirus isolates in different laboratories are not fully standardized and some might not be suitable to detect certain enterovirus serotypes (Harvala et al., 2018). Our method involves NGS analysis of whole capsid PCR products producing nucleotide sequence information at the serotype and strain level. We predict that our PCR method can amplify EV strains from all serotypes of all four A to D species.

While the methods presented here are robust and suitable for the characterization of EV genotypes directly from primary samples it is important to highlight potential limitations inherent to the approach taken. Targeted sequencing methods will always have the risk of missing some strains due to mismatches in primer binding sequence regions. Amplification-based methods, as used here both during the RT-PCR and sequencing library preparation steps, may also contribute to bias toward dominant species. However, we have so far been able to sequence EV strains from 101 (of the 110 different EV serotypes that have been described) obtained from different sources, including clinical isolates of EV-C104, EV-105, and EV-109 serotypes (unpublished data). Discerning viral quasispecies and genotypes with low abundance from sequencing noise is another limitation that should be taken into consideration.

While the method used here allowed detection of EV strains from all A, B, C, and D species we wanted to highlight the presence of EV-D68 and C109 serotypes because of their clinical relevance and the fact that they are known to be mainly respiratory viruses not commonly present in stool samples (Van Leer-Buter et al., 2016; Barnadas et al., 2017), so finding them in sewage was somehow unexpected. However, the presence of EV-D68 and EV-C109 in sewage has been reported before in Israel, Netherlands, and the United States (Benschop et al., 2017; Brinkman et al., 2017; Weil et al., 2017). It is possible that these serotypes are only excreted in low titers or for short periods of time in fecal samples. It is also important to consider that sewage might not only represent human urine and feces secretions but also wastewater from showering and other sources containing representatives of the human microbiome and virome. The EV-D68 sewage strain from Scotland clustered within genetic subclade B3 and preceded in time a large number of reported 2016 clinical isolates from Europe and the United States that were genetically related (Pariani et al., 2017; Wang et al., 2017), which demonstrates the value of ES for the early detection of EV strains potentially causing outbreaks. The EV-C109 sewage strain from Scotland was genetically very close to EV-C109 strains found in the same year in clinical samples from Denmark and Netherlands which further demonstrates the widespread circulation of this serotype in Europe.

Enterovirus strains from only 4 (E-11, CV-B2, E-6, and CV-B5) of the 13 serotypes found in the sewage concentrate, all from species B and closely related to EV strains present in the sewage concentrate, were found in cells infected with this concentrate. Although E-11, E-6, and CV-B5 strains were the most prevalent in the sewage concentrate, other EV-A, EV-C, and EV-D, strains, such as CV-A16, CV-A22, EV-C109, and EV-D68, were present in relatively high frequency but were not found in the infected cell extract. Clinical virus isolates from all four EV species have been shown to infect RD cells (Fernandez-Garcia et al., 2017), but high sensitivity for infection with species B EVs has been reported before (Faleye and Adeniji, 2015). EV-B strains in sewage concentrates would likely outcompete viruses from other species when growing on RD cells. This has important implications for EV molecular diagnosis as using this cell line, commonly done in clinical laboratories, restricts the number of EV serotypes that can be identified in clinical samples. This restriction is more relevant when analyzing environmental samples as they are more likely to contain complex EV mixtures.

## Conclusion

In conclusion, we have identified and characterized multiple EV strains in a sewage sample from Scotland using NGS analysis of PanEV-specific RT-PCR products obtained directly from the sewage concentrate, including EV strains from EV-C109 and EV-D68 serotypes typically found in respiratory samples. We show how ES in combination with NGS analysis can produce detailed genetic information of multiple EV strains in mixtures contributing to the detection of EV strains of public health relevance that could potentially cause outbreaks. The use of this approach will result in a significant advance in the molecular diagnosis of EVs and will help us improve our understanding of EV circulation patterns in human populations.

## Author Contributions

MM and JM designed the study. MM performed the practical laboratory work. JM analyzed the data and wrote the manuscript. MM and JM reviewed the manuscript and wrote the final version.

## Conflict of Interest Statement

The authors declare that the research was conducted in the absence of any commercial or financial relationships that could be construed as a potential conflict of interest.

## Footnotes

1 http://tree.bio.ed.ac.uk/software/tracer/

2 http://beast.bio.ed.ac.uk/treeannotator

3 http://tree.bio.ed.ac.uk/software/figtree/
